# Enhanced treatment of landfill leachate by biochar-based aerobic denitrifying bacteria functional microbial materials: Preparation and performance

**DOI:** 10.3389/fmicb.2023.1139650

**Published:** 2023-02-08

**Authors:** Jianyang Song, Minghui Li, Chunyan Wang, Yujie Fan, Yuan Li, Yongkun Wang, Wenxiao Zhang, Haisong Li, Hongyu Wang

**Affiliations:** ^1^Henan Key Laboratory of Industrial Microbial Resources and Fermentation Technology, Nanyang Institute of Technology, Nanyang, China; ^2^School of Civil Engineering, Nanyang Institute of Technology, Nanyang, China; ^3^School of Civil Engineering, Wuhan University, Wuhan, China; ^4^College of Ecology and Environment, Zhengzhou University, Zhengzhou, China

**Keywords:** aerobic denitrifying bacteria, landfill leachate, immobilization, sodium alginate, biochar, polyvinyl alcohol

## Abstract

**Objective:**

In this work, polyvinyl alcohol (PVA) and sodium alginate (SA) were used as entrapped carriers and *Artemisia argyi* stem biochar (ABC) was used as an absorption carrier to immobilize aerobic denitrifying bacteria screened from landfill leachate, thus a new carbon-based functional microbial material (PVA/SA/ABC@BS) was successfully prepared.

**Methods:**

The structure and characteristics of the new material were revealed by using a scanning electron microscope and Fourier transform infrared spectroscopy, and the performance of the material for treating landfill leachate under different working conditions was studied.

**Results:**

ABC had abundant pore structures and that the surface contained many oxygen-containing functional groups, carboxyl groups, and amide groups, etc. and it had good absorbing performance and strong acid and alkali buffering capacity, which was beneficial to the adhesion and proliferation of microorganisms. After adding ABC as a composite carrier, the damage rate of immobilized particles was decreased by 1.2%, and the acid stability, alkaline stability, and mass transfer performance were increased by 9.00, 7.00, and 56%, respectively. When the dosage of PVA/SA/ABC@BS was 0.017g/ml, the removal rates of nitrate nitrogen (NO_3_^−^-N) and ammonia nitrogen (NH_4_^+^-N) were the highest, which were 98.7 and 59.4%, respectively. When the pH values were 11, 7, 1, and 9, the removal rates of chemical oxygen demand (COD), NO_3_^−^-N, nitrite nitrogen (NO_2_^−^-N) and NH_4_^+^-N reached the maximum values, which were 14.39, 98.38, 75.87, and 79.31%, respectively. After PVA/SA/ABC@BS was reused in 5 batches, the removal rates of NO_3_^−^-N all reached 95.50%.

**Conclusion:**

PVA, SA and ABC have excellent reusability for immobilization of microorganisms and degradation of nitrate nitrogen. This study can provide some guidance for the great application potential of immobilized gel spheres in the treatment of high concentration organic wastewater.

## 1. Introduction

At the present stage, about 50 million tons of landfill leachate is produced every year in China. Generally, leachate is black or tawny, and it mainly contains water leaked from biochemical reactions of garbage during stacking and landfill processes and its inherent water (Dadrasnia et al., 2017; Song et al., 2019a). The landfill leachate contains a lot of organic matters which are difficult to degrade, heavy metal ions, ammonia nitrogen (NH_4_^+^-N), and various toxic and harmful pollutants (Song et al., 2020). Usually, leachate is toxic and poses a potential threat to the surrounding environment and the ecosystem (Baderna et al., 2019; Jagaba et al., 2021). Due to the complexity of landfill leachate, it is very difficult for the landfill to treat leachate, and therefore a proper treatment process shall be selected according to the composition and characteristics of the leachate (Costa et al., 2019). As the treatment of landfill leachate with a traditional process of wastewater treatment was not satisfactory, a method for treating leachate must be developed, implemented, and enhanced to treat a large amount of leachate effectively.

For the treatment of landfill leachate, a variety of technologies, which include biological treatment such as activated sludge and fluidized bed reactor process, chemical treatments such as Fenton process and chemical precipitation, and physical–chemical treatments such as absorption and membrane process to treat landfill leachate, were developed by the researchers (Teng et al., 2021). Compared to the high cost, high requirements, sensitive operating conditions, and a large amount of excess sludge of these methods, biological enhancement is known as a cost-effective choice for treating high concentration organic wastewater (Abuabdou et al., 2020; Wang et al., 2021; Xu et al., 2021). When specific functional bacteria are used for biological enhancement of high concentration organic wastewater treatment systems, free-degrading bacteria have a variety of defects, such as low mechanical strength, low cell density, difficulty in separating biomass effluents, susceptibility to water flow patterns, and limitation in application of practical leachate treatment (Mulla et al., 2013). Using immobilized microorganism technology to immobilize water treatment microorganism on the carrier is to restrict microorganism with specific functions to specific limited space to facilitate recycling and maintain stable biological activity, reduce biological loss, enhance the resistance to harsh environment, and improve the efficiency of wastewater treatment (Qiao et al., 2010; Chen et al., 2021). Microorganisms can be adsorbed on the surface of the porous structure by inorganic carrier materials such as activated carbon and biochar. Hydrogel is a three-dimensional flexible polymer formed by chemical and physical crosslinking of synthetic or natural materials, which adsorbs pollutants on its surface and in the three-dimensional network (Fang et al., 2016; Khan and Lo, 2016). In terms of wastewater treatment, hydrogel has good pollutant adsorption capacity, water absorption capacity, and reversible swelling capacity (Khan and Lo, 2017; Yang et al., 2021). Research shows that hydrogel is superior to traditional adsorbent in adsorption capacity (Zhu et al., 2016). Biochar is a new adsorption material, which is decomposed from carbon-rich biomass under oxygen-limited pyrolysis conditions which are generally lower than 700°C (Mohan et al., 2014). It is an excellent inorganic carrier, which can effectively adsorb microorganisms and provide a stable environment to maintain cell viability, thereby improving the efficiency of biological degradation (Liu et al., 2012). Previous studies have shown that *Artemisia argyi* stem biochar has a large number of mesopore and micropore structures, which can provide abundant attachment sites for microorganisms (Song et al., 2019b). Therefore, in more and more studies, immobilized microbial technology has been introduced into wastewater treatment. Zhang et al. (2021) successfully prepared alginate-based porous nanocomposite hydrogels to enhance the removal of Cr (VI) and Cu (II). Gan et al. (2018) used eucalyptus leaves as an adsorption carrier to immobilize *Burkholderia cepacia* so that the adsorption capacities of the immobilized microbial carrier on malachite green and Cr^6+^ were 68.60 and 47.50% higher than those of single bacteria, respectively.

Currently, it has become a research hotspot to use hydrogel immobilized microorganisms in the fields such as biology and environment, and using biochar as the adsorption carrier of microorganism has a wide application prospect. In most studies, microorganisms were immobilized on a single carrier while few articles focused on the immobilization of microorganisms, with hydrogel as the embedding carrier and biochar as the adsorption carrier, and they were used to treat landfill leachate. Therefore, the purposes of this work are as follows: (1) immobilize functional bacteria screened (BS) from the landfill leachate to prepare a new carbon-based functional material (PVA/SA/ABC@BS) for the treatment of leachate, using *Artemisia argyi* stem biochar (ABC) as an absorption carrier and using polyvinyl alcohol (PVA) and sodium alginate (SA) as entrapped carriers; (2) research the effect of hydrogel added with biochar on the physical–chemical properties of immobilized gel beads, including chemical stability, mechanical strength, and mass transfer; and (3) explore the decontamination performance and mechanism of PVA/SA/ABC@BS under different working conditions, and evaluate its reusability.

## 2. Materials and methods

### 2.1. Sample sources

In this work, the fresh leachate was collected from a landfill in Nanyang City and activated sludge was collected from an aerobic biological reactor. The samples were stored in sterilized centrifuge tubes and frozen on site. After the samples were taken back to the laboratory, the experiment was carried out immediately. The characteristics of leachate are shown in Table 1.

**Table 1:** Characteristics of landfill leachate.

pH	COD/(mg/L)	NH_4_^+^-N/(mg/L)	NO_3_^−^-N/(mg/L)	NO_2_^−^-N/(mg/L)	Color	Dark brown
7.30	7,175	1313.18	67.40	2.14	Dark brown	Strong

Values are given as an average.

### 2.2. Screening and identification of bacteria

Medium(g/L): (1) aerobic denitrification solid medium (DM): potassium nitrate 1.00; sodium succinate 16.67; disodium hydrogen phosphate 1.00; potassium dihydrogen phosphate 1.00; magnesium sulfate heptahydrate 0.15; microelement 2.00 ml; agar 20.00; pH 7.0 ~ 7.5; (2) bacteria strain fluid medium (FM): peptone 10.00; beef extract 3.00; sodium chloride 5.00; pH 7.4 ± 0.2; (3) bromothymol blue solid medium (BTB): potassium nitrate 1.00; sodium succinate 16.67; disodium hydrogen phosphate 1.00; potassium dihydrogen phosphate 1.00; magnesium sulfate heptahydrate 0.15; microelement 2.00 ml/L; BTB (0.10 g of bromothymol blue dissolved in 10.00 ml of ethanol) 2.00 ml; agar 20.00; pH 7.0 ~ 7.5;(4) composition of microelement solution: sodium molybdate tetrahydrate 0.02; anhydrous cupric sulfate 0.03; manganese chloride tetrahydrate 0.10; zinc sulfate 0.04; calcium chloride dihydrate 0.11; ferrous sulfate heptahydrate 0.10; ethylene diamine tetraacetic acid disodium 1.00; and cobalt chloride hexahydrate 0.03. The sterilization requirement for the culture medium was sterilization at 121°C for 15 min.

The activated sludge was made into sludge suspension. Sludge suspension (5 ml) was poured into 45 ml of DM medium for constant temperature shaking enrichment culture at 30°C with a speed of 160 r/min for 24 h. Enriched bacterial solution (5 ml) was poured into sterilized fresh DM medium and repeated for 4 times. Enriched bacterial solution (0.1 ml) was poured into 0.9 ml of deionized water to obtain 10^−1^ gradient bacterial solution, and this was repeated for 5 times to obtain 6 groups of gradient bacteria solution. Bacterial solution (0.1 ml) in each gradient group was coated on BTB solid medium, and three parallel groups were set for each gradient. After the bacterial solution was cultivated in the incubator at 40°C for 1 day, single colony with blue halo appeared in the medium. A sterilized inoculation ring was used to pick the single colonies of different shapes and sizes which were separated and purified with streaked lines on DM solid medium for several times, and the separated bacterial strain was evaluated for removal performances of nitrate, ammonia nitrogen, and COD.

16S rDNA gene sequencing was performed on the strains (Ren et al., 2014). The DNA of the strain was extracted as a PCR template for amplification, and the 16S rDNA gene universal primer 27F/1492R was used for PCR amplification. Conditions for PCR amplification were as follows: pre-denaturation at 98°C for 2 min, denaturation at 98°C for 10 s, annealing at 55°C for 15 s, extension at 72°C for 15 s, 35 cycles in total, and extension at 72°C for 5 min. The sequencing was performed by Beijing Qingke Biotechnology Co., Ltd., and the results were analyzed and compared with a software Blast.

### 2.3. Preparation of immobilized beads

The process for preparing biochar was based on the results of previous research (Song et al., 2019b). PVA (1 g) was placed in a beaker containing 100 ml ultra-pure water in it. Then, the beaker was placed on the electronic multipurpose stove to dissolve PVA completely into transparent PVA solution. And then, 1 g of SA was added to a conical flask containing 100 ml of PVA solution. Next, the conical flask was stirred and shaken in a shaker whose speed is 120 r/min for 24 h until SA was fully dissolved to obtain white mixture A. ABC of different masses (1, 2, and 3 g) was added to the mixture A and the mixture was stirred with a glass stick so that ABC was completely mixed with the mixture A to obtain a black mixture (B, C, and D) of the same viscosity. The mixture was drawn with a 5 ml syringe and added droplet by droplet to a continuously stirred 300 mLCaCl_2_ solution (5%, w/v) to cross-link to form gel balls, which were further hardened for 24 h. And next, the gel beads were repeatedly rinsed with deionized water to wash away the residual Ca^2+^ on the surface to obtain PVA/SA gel beads (or PVA/SA/ABC gel beads).

Two hundred PVA/SA/ABC gel beads were placed in a conical flask containing 100 ml of bacterial suspension and the beads were completely immersed in the suspension. The conical flask was sealed with a sealing film and placed in a shaker (37°C, 160 r/min) for continuous shaking for 48 h, and then the beads were taken out from the flask and marked as type I PVA/SA/ABC@BS gel beads. Bacterial suspension (30 ml) was injected into mixture C, and a glass stick was used to stir the mixture. After it was mixed evenly, a 5 ml syringe was used to prepare the mixture into immobilized beads. The method was the same as that described above, and the beads were marked as type II PVA/SA/ABC@BS gel beads. The preparation process of the immobilized beads and batch influence experiments were conducted in the sterile operating table.

### 2.4. Experiment design and operating parameters

In order to research the influence of ABC dosage on physical and chemical properties of PVA/SA/ABC gel beads, the dosages of ABC were 1, 2, and 3 g, respectively. Under the conditions that the reaction temperature was 25°C, that the dosage of PVA/SA/ABC@BS gel beads was 0.01 g/ml, and that the leachate was diluted 5 times, the influence of different working conditions on the decontamination performance of PVA/SA/ABC@BS gel beads was studied. In order to research the reusability of immobilized beads, the immobilized beads were recycled at the end of each experiment. After being washed with 0.85% sodium chloride solution, the beads were reused for leachate treatment.

### 2.5. Analytical methods

Determination of mechanical strength: a certain amount of gel beads were placed in a conical flask containing 100 ml of wastewater. After being sealed with a sealing film, the flask was placed in a constant temperature shaker (180 r/min) for continuous shaking for 48 h. The damage condition was recorded. The ratio of the number of apparently incomplete particles to the number of beads initially placed in the conical flask was recorded as the breakage rate.

Determination of mass transfer performance: the gel beads were placed into a 10 mm colorimetric dish and soaked with 5 ml of blue ink so that the blue ink completely covered the gel beads. After 8 min, the gel bead was cut along its center with a knife, and the radius of the ink immersed into the center of the bead was measured and recorded. The ratio of the immersed radius by blue ink to the radius of the bead was recorded as mass transfer rate.

Determination of chemical stability: a certain amount of gel beads were placed in a conical flask containing 100 ml mixture of HCl solution and NaOH solution, the concentration of the HCl solution was 1 mol/L while the pH was 4, and pH of the NaOH solution was 10. After 72 h, the gel beads were taken out of the conical flask to observe the dissolution, damage, and loose structures. The ratio of the number of apparently incomplete particles to the number of beads initially placed in the conical flask was recorded as the deformation rate.

The method for determining yields of biochar was referred to the method described in the previous literature (Song et al., 2019b). The surface of the material was observed and measured by a scanning electron microscope (SEM, JSM-7900F, Japan). The functional groups on the surface of the material were qualitatively analyzed by Fourier transform infrared spectroscopy (FTIR, Thermo Nicolet, 6700, Madison, WI, United States).

After the supernatant of the reaction system was filtered by the 0.45 μm microporous membrane, COD, NH_4_^+^-N, NO_2_^−^-N, and NO_3_^−^-N in the filtrate were measured with standard methods (Apha., 2012). The pH values were measured with a pH meter (pHS-25, Shanghai Leici Instrument Factory, China).

### 2.6. Statistical analysis

All statistical analyses were performed using SPSS version 15.0. Pair-sample *t*-tests were used to assess whether the water quality and the physical and chemical properties of immobilized pellets were significantly different between samples based on *p*-values. A *p*-value of <0.05 was considered significant.

## 3. Results and discussion

### 3.1. Identification results and performance of the strains

After separation and purification, 7 strains of aerobic denitrifying bacteria were successfully screened on DM medium and named as D00#, D01#, D02#, D03#, D04#, D05#, and D06#, respectively. With 16S rDNA sequence alignment, the identification results are shown in Table 2.

**Table 2:** Screening results of aerobic denitrification functional bacteria.

Strain number	Strain name	Morphological description	Similarity (%)
D00#	*Pseudomonas yangonensis*	White, large colony, flat, dry	99.54
D01#	*Acinetobacter*	Off-white, round, opaque	99
D02#	*Pseudomonas alcaliphila*	Off-white, dry, regular edges, flat	100
D03#	*Pseudomonas oleovorans*	Partial transparent, center bulge, dry, large colony	99.69
D04#	*Paracoccus versutus*	White, raised, wet	100
D05#	*Pseudomonas stutzeri*	White center, transparent around, dry, wet	99.85
D06#	*Paracoccus communis*	Off-white, large colony, pustular, convex, viscous	100

**Figure 1:**
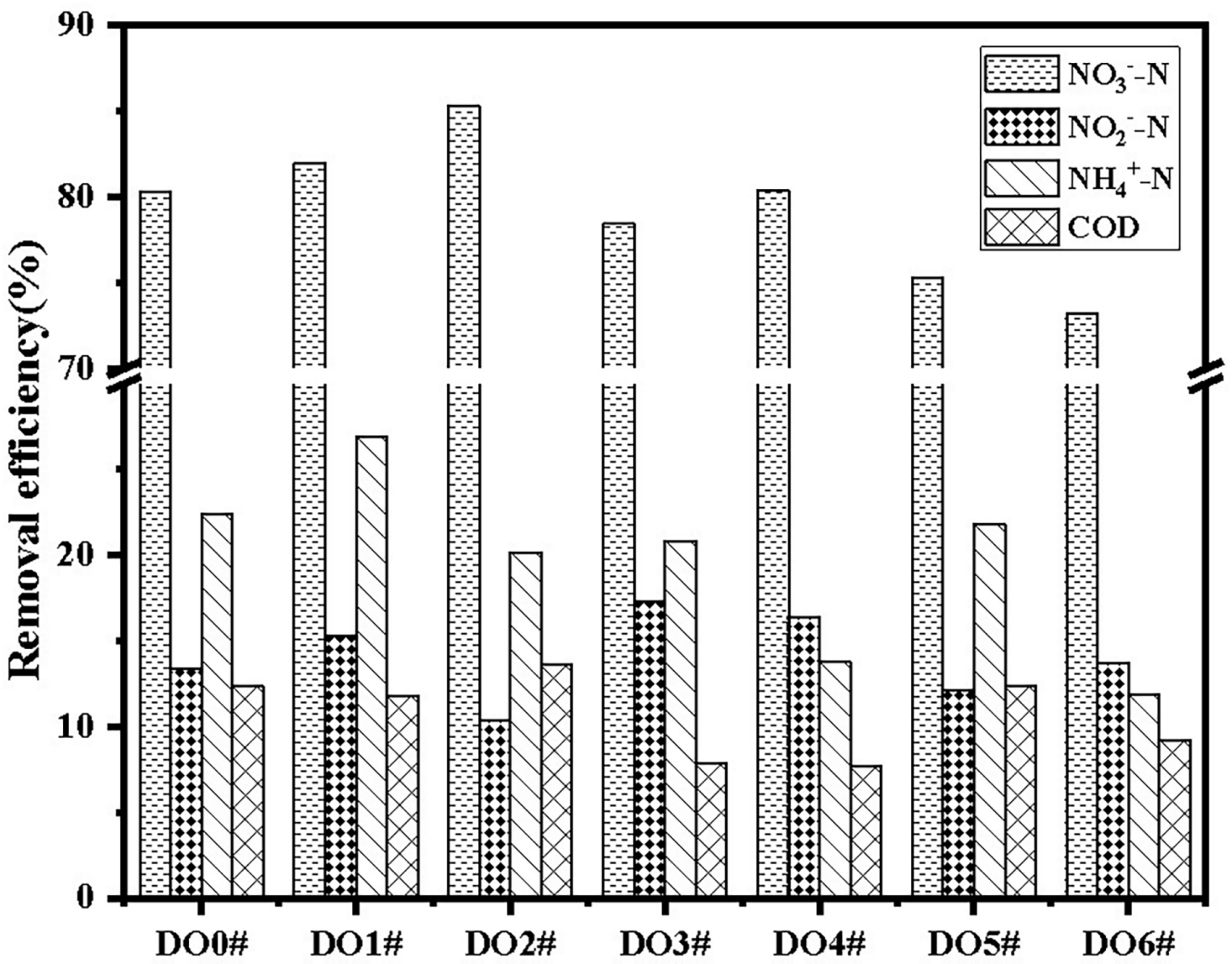
Performance of dominant bacteria.

As shown in Figure 1, D01# had the best aerobic denitrification performance (*p* < 0.05), and therefore, it was used in subsequent experiments and studies. According to the morphology and physiological characteristics of the strain and *Berger’s Manual of Bacterial Identification*, it was preliminarily identified to belong to *Acinetobacter*. Sequence alignment was performed to the strain D01# with 16S rDNA to obtain a sequence whose length was 1,500 bp. BLAST search in NCBI database showed that the homology was 99%, indicating that the strain D01# belonged to *Acinetobacter* sp.

The functional bacteria were aerobic denitrifying bacteria with favorable capacity of removing NO_3_^−^-N, and the removal rate reached 81.94%. The capacity of the strain to remove COD was attributed to the fact that denitrifying functional bacteria consumed carbon sources during the process of removing nitrate nitrogen (Yan et al., 2017).

### 3.2. Physical and chemical properties of PVA/SA/ABC@BS

**Figure 2:**
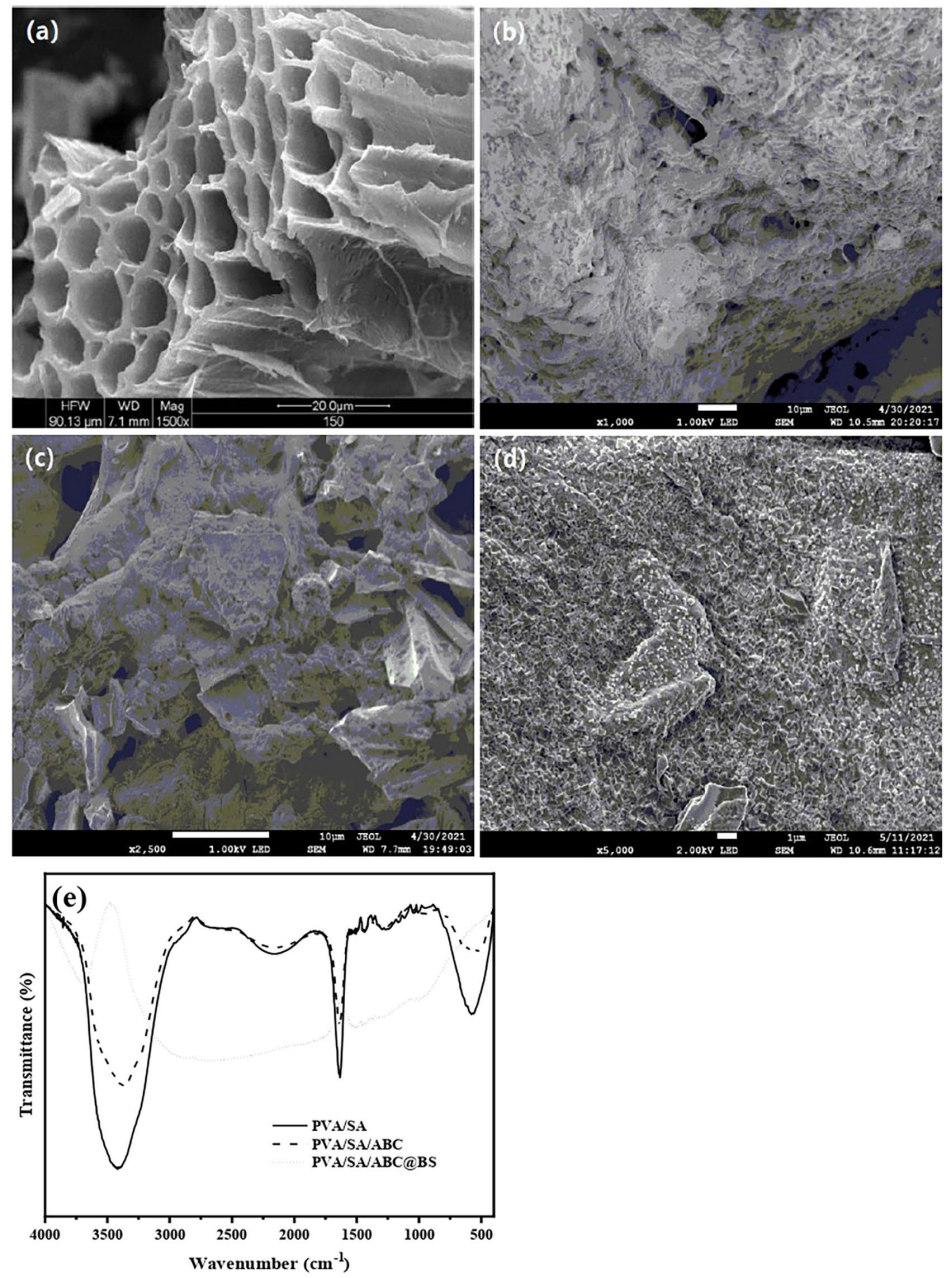
The SEM images of **(A)** ABC, **(B)** PVA/SA, **(C)** PVA/SA/ABC, **(D)** PVA/SA/ABC@BS and the FTIR spectra of different gel beads **(E)**.

In this work, the yield of ABC prepared at 600°C using *Artemisia argyi* stem as raw material was about 33.50% (w/w). Figure 2A shows that the internal structure of ABC consisted of multiple channels stacked on each other, that apertures of the channels were different, and that there were many small channels between the channels. The wall of the channel was smooth, but it had various pore structures. Pores of the biochar provided habitat for BS. The channels mainly provided space for proliferation of BS and also provided abundant carbon sources for BS directly. As shown in Figure 2B, PVA/SA gel materials were bonded closely to each other, and there were various types of pores between the materials, so that they have a certain specific surface area and provide space for BS loading, which is conducive to the diffusion of adsorbent molecules and adsorption to the internal active site. The inside of the bead was a honeycomb structure with large porosities which could provide adequate microenvironment for growth of microorganisms and was conducive to the adhesion and proliferation of microorganisms. Figure 2C shows that the gel beads were well coupled with ABC, that the surface characteristics of the gel beads introduced with ABC changed obviously, and that many granular bulges of ABC were irregularly distributed on the surface structure without damaging the multi-channel structure of ABC. The numbers of pore structures and adsorption sites of the gel beads coupled with ABC increased, and the surface of ABC was no longer smooth after being bonded with abundant gel materials. Figure 2D shows that the surface characteristics of PVA/SA/ABC@BSgel beads introduced with BS changed obviously. As BS was adsorbed by small pores on the surface of ABC and the gel material, it was difficult to see the pore structures of the gel material directly. Moreover, the shape of the cross section of ABC channel was greatly changed, and the surface of ABC was not smooth any more after being bonded with microorganisms. However, the multi-channel structure of ABC was not damaged.

Figure 2E shows that representative characteristic absorption peaks of O-containing functional groups of PVA/SA gel beads were at about 3,418 cm^−1^ and belonged to the stretching vibration of -OH, which meant that there was a hydroxyl group in the molecule (Song et al., 2020). In case there was a strong absorption peak near 1,658 cm^−1^, it meant that there was a carboxyl group in the molecule. These oxygen-containing functional groups were conducive to the adsorption of pollutants. After the use of ABC, the types of functional groups on the surface of the gel materials did not change a lot. That was because a large amount of hydrogels were bonded to the surface of ABC, so that the material mainly showed the functional groups of PVA/SA gel beads. The representative characteristic adsorption peaks such as C-containing functional groups of PVA/SA/ABC gel beads were at about 3,372 cm^−1^ and belonged to the stretching vibration of -C ≡ C-H; If the peak was near 1,633 cm^−1^, it belonged to -N=N = vibration. As the C=O stretching vibration of the amide band of solid substance and the deformation vibration of NH_2_ were both near 1,650 cm^−1^, biochar might contain amide groups (Figure 2E). The surface functional groups of the PVA/SA/ABC@BS introduced with microorganisms were significantly changed due to the proliferation of BS in the pores of the composites and the adsorption sites of the above-mentioned functional groups. The representative characteristic adsorption peaks such as C-containing functional groups were at about 3,418 cm^−1^ and belonged to the stretching vibration of -C ≡ C-H (Figure 2E). The research showed that groups such as amides, amines, and carboxyls were beneficial to the adhesion and reproduction of cells (Lee et al., 1994). After the introduction of BS, BS was adsorbed on these functional groups and oxygen-containing functional groups so that types of functional groups on the surface of the composite material changed significantly. In the presence of carboxyl or amide functional groups, copper tetravalerate complex would be formed on the surface of hydrogel (Orozco-Guareño et al., 2010). It was speculated that it was suitable to use PVA/SA/ABC gel beads as microbial composite carriers.

**Figure 3:**
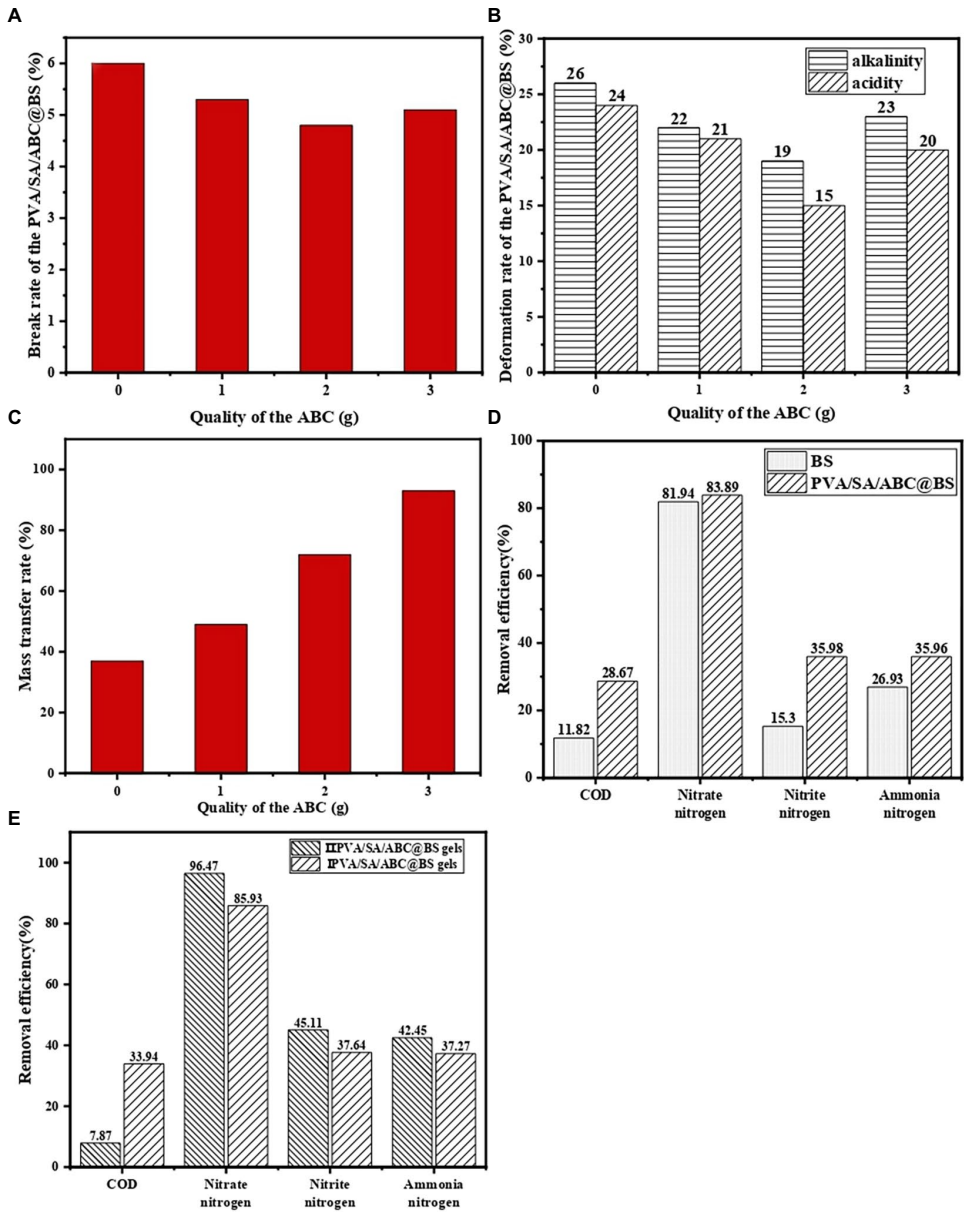
The **(A)** damage rate, **(B)** deformation rate, **(C)** mass transfer performance of PVA/SA/ABC@BS prepared with different dosages of ABC (the volume of the solution was 100 ml, and the dosage of PVA and SA was both 1 g) and the decontamination effect of different gel beads **(D,E)**.

Figure 3A shows the damage rate of PVA/SA/ABC@BS at different dosages of ABC. The results showed that PVA/SA gel beads had excellent mechanical properties and the damage rate was only 6.00%, which meant that it was suitable to use the embedding material in the process of high hydraulic load. The dosage of ABC had some influence on the mechanical properties of the composite material, but the influence was not significant. After ABC was added, the damage rate of gel beads was decreased and the mechanical stability of gel beads was strengthened. However, the damage rate was increased again when the mass of ABC added reached 3 g. This might be due to the fact that the proportion of biochar in the material was large and the number of channels and pore structures in the material was increased after more biochar powders were added. After being stirred adequately, the material was easy to separate and dissolve and therefore, its damage rate was increased. Meanwhile, the mass fraction of SA had an important influence on the mechanical strength of the material. When the proportion of SA in the material was decreased, the tensile strength of the gel fiber was small, so that the damage rate was increased. When the mass of ABC added was 2 g, the damage rate was 4.80%. Compared with the group without adding ABC, the damage rate was decreased by 1.20%. In water treatment, the mechanical properties of the material played an important role in process selection and economic performance. Using material with strong mechanical properties could avoid the dispersion of dominant bacteria and ensure the stability of the treatment process.

Figure 3B shows deformation rate of PVA/SA/ABC@BS prepared with different dosages of ABC under acid and alkali conditions. The research showed that PVA/SA/ABC@BS had a smaller deformation rate under acidic conditions if the mass fraction of ABC in the prepared material was the same. The gel beads were more stable under acidic conditions. It was probably because the functional groups on the surface of the gel materials were more likely to combine protons to form stable structures. Moreover, with the increase of the mass fraction of ABC, the deformation rate of PVA/SA/BC gel beads was gradually decreased. After the mass of ABC reached 3 g, the deformation rate was increased. When the mass of ABC added was 2 g, the damage rates at alkaline and acidic conditions were 19.00 and 15.00%, respectively. The damage rates were decreased by 7.00 and 9.00%, respectively, compared with those without adding ABC (*p* < 0.05). Therefore, the gel materials introduced with ABC had higher chemical stability.

Figure 3C shows the mass transfer performance of PVA/SA/ABC@BS prepared with different dosages of ABC. The results showed that the mass transfer performance of PVA/SA/BC was proportional to the mass of ABC mixed in the material. PVA/SA gel beads had certain mass transfer performance, and the mass transfer rate was 37.00%. If 3 g of biochar was added into the material, the mass transfer rate of PVA/SA/BC could reach 93.00%, which was increased by 56.00% (*p* < 0.05). After the PVA/SA/BC were dipped into the ink, the ink diffused from the surface of the beads to the center of the beads, which accorded with the preconceived results of the experiment. This might be due to the fact that the introduction of biochar provided more channel space for the transfer of ink, resulting in a faster mass transfer rate and a wider range of mass transfer. The mass transfer performance of composite materials could guarantee the proliferation of microorganisms and the adsorption of pollutants.

Figure 3D shows a comparison between the decontamination effect of a single bacterial colony and that of PVA/SA/ABC@BS. The results showed that the decontamination efficiency of the single bacterial colony was lower than that of PVA/SA/ABC@BS and that the effects of NO_3_^−^-N removal were similar, which proved that NO_3_^−^-N in water was mainly removed by aerobic denitrification of functional bacteria. However, there was a gap in the removal rates of other indexes, indicating that embedding carrier could adsorb some pollutants in water.

In summary, *Artemisia argyi* stem biochar had abundant pore structures and contained many oxygen-containing functional groups, carboxyl groups, hydroxyl groups, and amide groups on its surface. Moreover, it had excellent adsorption performance and strong acid and alkali buffering capacity, which was beneficial to the adhesion and proliferation of microorganisms. The damage rate of the immobilized beads was reduced by 1.20%, and the acid stability, alkaline stability, and mass transfer were increased by 9.00, 7.00, and 56.00%, respectively, by adding *Artemisia argyi* stem biochar as a composite carrier. Therefore, PVA/SA/ABC@BS had good physical and chemical properties for landfill leachate treatment, and batch experiments will be conducted to determine the optimal operating conditions.

### 3.3. Influence of preparation methods on decontamination performance of PVA/SA/ABC@BS

The influence of preparation methods on decontamination performance of PVA/SA/ABC@BS was studied at the conditions that the reaction temperature was 25°C, that the dosages of type I and type II gel beads were both 0.01 g/ml, that the leachate was diluted 5 times, and the reaction time was 96 h. Figure 3E shows that the removal rates of NO_3_^−^-N, NO_2_^−^-N, and NH_4_^+^-N in leachate by type II PVA/SA/ABC@BS gel beads were slightly higher than those by type I PVA/SA/ABC@BS gel beads. The removal rate of COD by type II PVA/SA/ABC@BS gel beads was much lower than that by type I PVA/SA/ABC@BS gel beads. In general, the growth and reproduction of BS on type II PVA/SA/ABC@BS gel beads were better. It might be because the long-term coupling made microorganisms have certain adaptability to the internal material and certain tolerance to the external environment. No matter which method was adopted to prepare immobilized beads, the removal rate of NO_3_^−^-N was the highest, followed by that of NO_2_^−^-N. However, the removal rate of NO_2_^−^-N was lower than 50%, while the removal rate of COD was the lowest. The reason might be that biochar could also provide a carbon source for microbial reproduction, thus reducing the consumption of COD in landfill leachate (*p* < 0.05). The preparation method had certain influence on the convenience, cycle, and treatment effect of water treatment, and therefore, it was necessary to constantly develop and select appropriate method for preparing materials in practical application. In this study, type II PVA/SA/ABC@BS gel beads were selected for subsequent batch experiments.

### 3.4. Influence of coupling time period on decontamination performance of PVA/SA/ABC@BS

**Figure 4:**
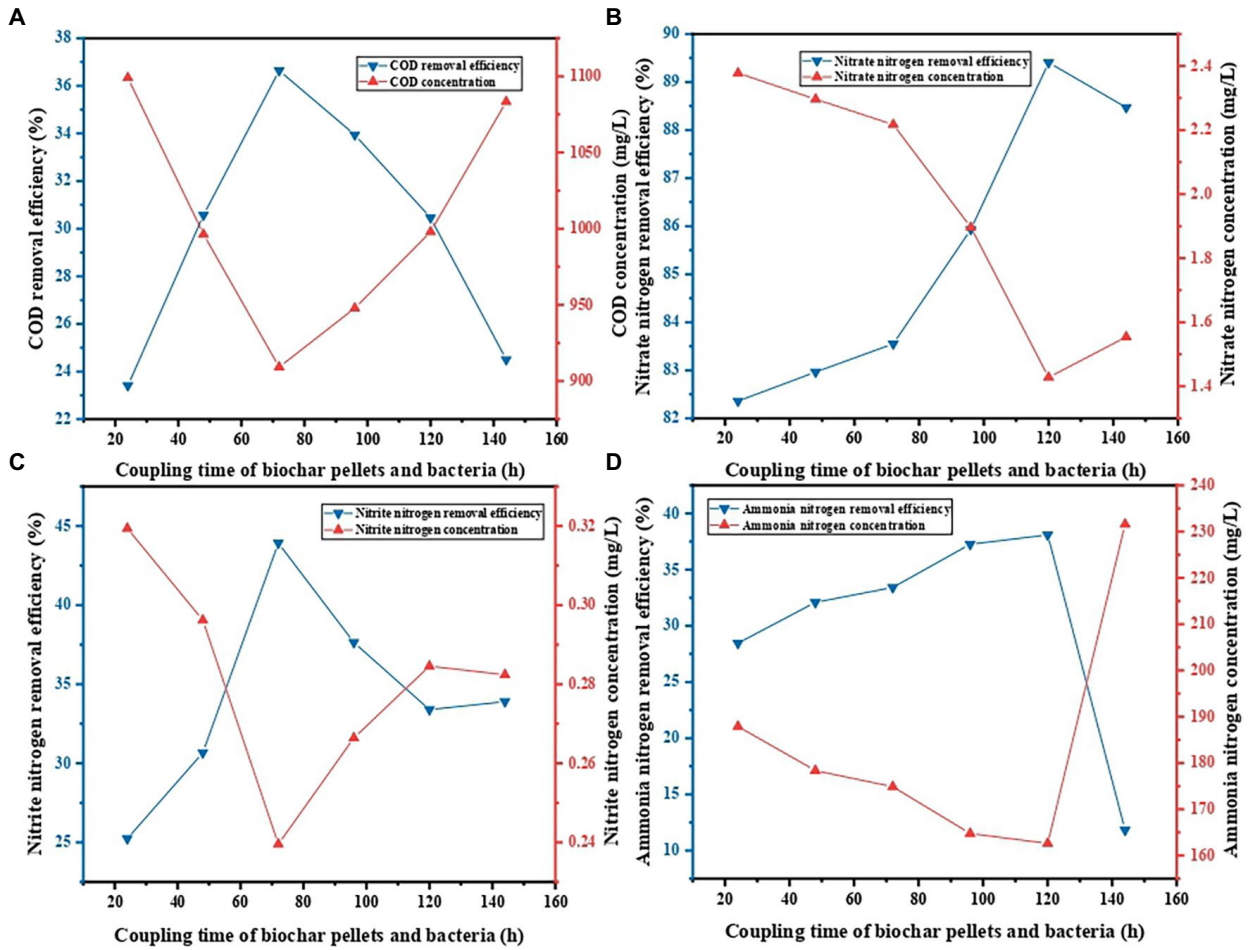
Effect of coupling time on contaminant removal: **(A)** COD removal, **(B)** NO_3_^−^-N removal, **(C)** NO_2_^−^-N removal, and **(D)** NH_4_^+^-N removal.

When the coupling time periods of BS and PVA/SA/ABC were 24, 48, 72, 94, 120, and 144 h, respectively, the decontamination performance of PVA/SA/ABC@BS was studied, respectively, under the following conditions: the reaction temperature was 25°C; the dosage of gel beads was 0.01 g/ml; the leachate was diluted 5 times; and the reaction time was 96 h. Figure 4 shows that, under the same condition, the maximum removal rate of COD was 36.64% when the coupling time period was 72 h, and that the minimum value was 23.41% when the coupling time period was 24 h. The maximum removal rate of NO_3_^−^-N was 89.41% when the coupling time was 120 h and the minimum value was 82.36% when the coupling time period was 24 h. The maximum removal rate of NO_2_^−^-N was 43.92% when the coupling time period was 72 h and the minimum value was 25.23% when the coupling time period was 24 h. The maximum removal rate of NH_4_^+^-N was 38.08% when the coupling time period was 72 h and the minimum value was 31.13% when the coupling time was 144 h. The removal efficiency was increased with the increase of the coupling time period (*p* < 0.05). However, the removal efficiency was decreased to different degrees when the coupling time period was too long. The reason might be that the growth and proliferation of microorganisms inside the materials reached saturation and the microorganisms began to compete with each other when the coupling time period was too long, so that the activity of bacteria and the removal rate were both decreased. Different coupling time periods exerted some influence on the preparation cycle and the decontamination effect. The above experimental results indicated that the coupling time of BS and PVA/SA/ABC of 72 h is appropriate for the subsequent batch experiments.

### 3.5. Influence of dosage on decontamination performance of PVA/SA/ABC@BS

**Figure 5:**
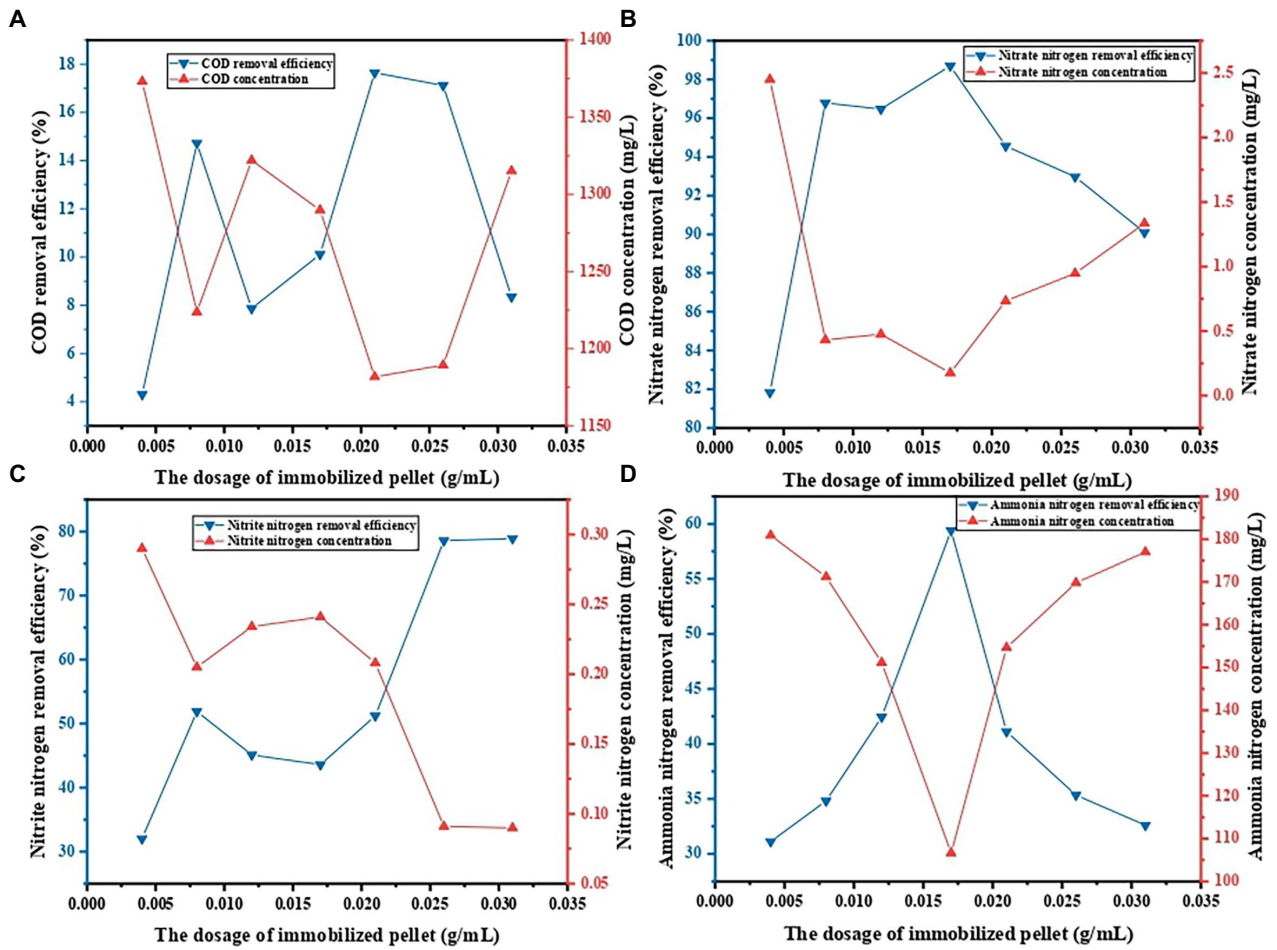
Effect of the dosage of immobilized pellet on contaminant removal: **(A)** COD removal, **(B)** NO_3_^−^-N removal, **(C)** NO_2_^−^-N removal, and **(D)** NH_4_^+^-N removal.

The influence of dosage on decontamination performance of PVA/SA/ABC@BS was studied under the conditions that the reaction temperature was 25°C, that the leachate was diluted 5 times, and that the reaction time was 96 h. Figure 5 shows that, under the same condition, the maximum removal rate of COD was 17.65% when the dosage was 0.021 g/ml, and the minimum value was 4.30% when the dosage was 0.004 g/ml. The maximum removal rate of NO_3_^−^-N was 98.70% when the dosage was 0.017 g/ml, and the minimum value was 81.83% when the dosage was 0.004 g/ml. The maximum removal rate of NO_2_^−^-N was 78.93% when the dosage was 0.0312 g/ml, and the minimum value was 32.03% when the dosage was 0.004 g/ml. The maximum removal rate of NH_4_^+^-N was 59.40% when the dosage was 0.017 g/ml, and the minimum value was 31.13% when the dosage was 0.004 g/ml. Except for NO_2_^−^-N, the removal rates of other indexes were all decreased when the dosages were too high or too low (*p* < 0.05). However, the removal rate of NO_2_^−^-N reached saturation when the dosage reached 300. The reason might be that the initial concentration of NO_2_^−^-N in the water sample was extremely low, and BS was aerobic denitrifying bacteria, which will not cause the accumulation of NO_2_^−^-N in the system. However, other indexes showed high removal rates if appropriate dosages were used. It might be because the amount of BS in the water sample was low and the concentration of pollutants in the water sample was too high when the dosage was small, resulting in insufficient activity of microorganisms. When the dosage was too large, the competition among microorganisms was formed and the adsorption sites in the adsorbent were not effectively utilized when the dosage was too high. Therefore, under the experimental conditions, when the dosage of PVA/SA/ABC@BS was 0.017 g/ml, it is conducive to the synchronous removal of pollutants in landfill leachate.

### 3.6. Influence of concentration of pollutant on the decontamination performance of PVA/SA/ABC@BS

**Figure 6:**
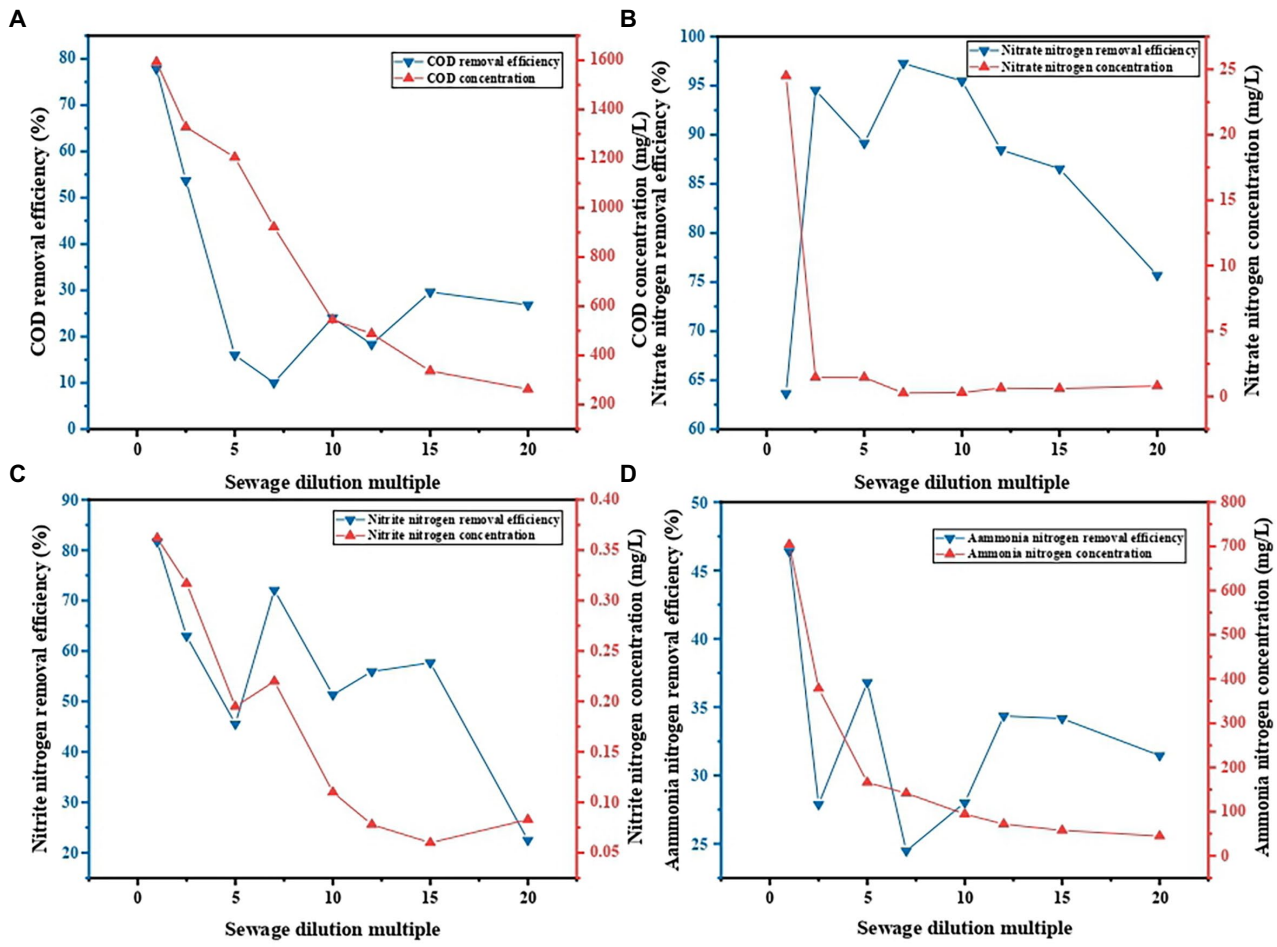
Contaminant removal under different wastewater concentrations: **(A)** COD removal, **(B)** NO_3_^−^-N removal, **(C)** NO_2_^−^-N removal, and **(D)** NH_4_^+^-N removal.

The influence of concentration of pollutant on decontamination performance of PVA/SA/ABC@BS was studied under the conditions that the reaction temperature was 25°C, that the dosage of gel beads was 0.01 g/ml, and that the reaction time was 96 h. Figure 6 shows that, under the same condition, the maximum removal rate of NO_3_^−^-N was 97.28% when the concentration of NO_3_^−^-N in wastewater before treatment was 9.63 mg/L and the minimum value was 63.65% when the concentration of NO_3_^−^-N in wastewater before treatment was 67.40 mg/L. The maximum removal rate of NO_2_^−^-N was 81.65% when the concentration of NO_2_^−^-N in wastewater before treatment was 2.14 mg/L and the minimum value was 22.51% when the concentration of NO_2_^−^-N in wastewater before treatment was 0.11 mg/L. The removal rates of other indexes except for NO_3_^−^-N showed the highest levels when the concentration of pollutant was low, while the removal rate of NO_3_^−^-N showed the optimal level when the concentration of pollutant was moderate (*p* < 0.05). The reason might be that PVA/SA/ABC@BS was selective to NO_3_^−^-N with a certain concentration. When the concentration of NO_3_^−^-N was low, gel beads showed a certain degree of insensitivity to NO_3_^−^-N in the water sample, so the gel beads moved toward COD and NO_2_^−^-N. However, when the concentration of pollutants was high and within a certain range, PVA/SA/ABC@BS was still selective to NO_3_^−^-N. As the active sites of adsorbent were utilized to the maximum extent, the removal rate of NO_3_^−^-N was increased (Wu et al., 2020). However, when the concentration of pollutants was too high, the activity of microorganism showed fatigue after saturation was reached, and the excess pollutants could not be ingested, which resulted in a decrease in the removal rates of various indexes. The experimental results showed that too low pollutant concentration was not conducive to the removal of pollutants by PVA/SA/ABC@BS. Under the existing experimental conditions, it was appropriate to control the dilution ratio within 20.

### 3.7. Influence of initial pH value of the solution on the decontamination performance of PVA/SA/ABC@BS

**Figure 7:**
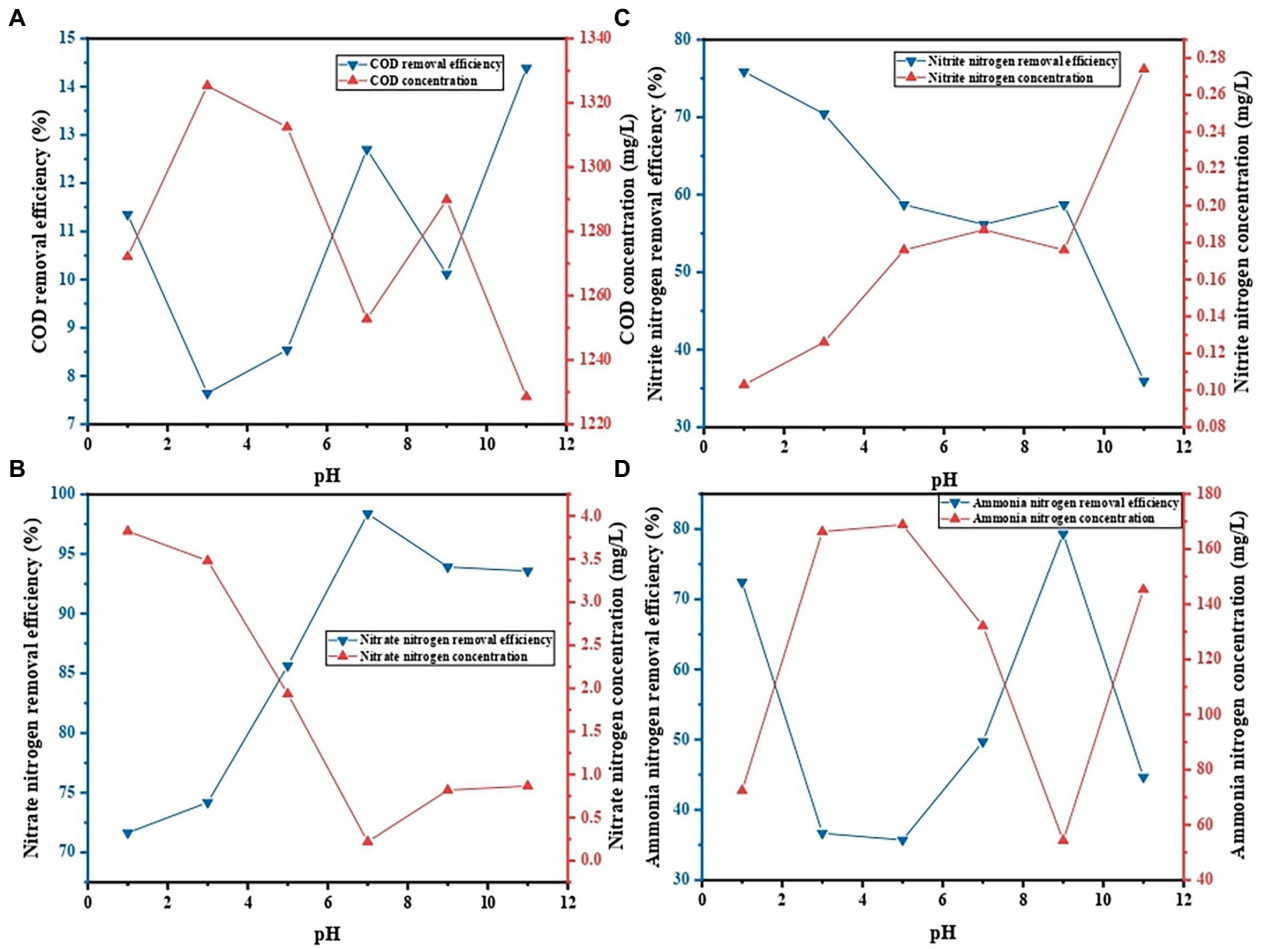
Effect of initial pH on contaminant removal: **(A)** COD removal, **(B)** NO_3_^−^-N removal, **(C)** NO_2_^−^-N removal, and **(D)** NH_4_^+^-N removal.

The pH values of the solution will affect the surface charge and status of the functional groups on the surface of the adsorbent, and thus affecting the types and chemical properties of the surface of the adsorbent (Ma et al., 2018). Therefore, in the actual treatment of wastewater, the influence of pH values on the decontamination performance of gel beads should be considered. The influence of the initial pH value of the solution on decontamination performance of PVA/SA/ABC@BS was studied under the conditions that the reaction temperature was 25°C, that the dosage of gel beads was 0.01 g/ml, and that the reaction time was 96 h. Figure 7 shows that, under the same condition, the maximum removal rate of NO_3_^−^-N was 98.38% when the pH value of the solution was 7, and the minimum removal rate was 71.64% when the pH value of the solution was 1 (*p* < 0.05). The maximum removal rate of NO_2_^−^-N was 75.87% when the pH value of the solution was 1, and the minimum removal rate was 35.94% when the pH value of the solution was 11 (*p* < 0.05). The maximum removal rate of NH_4_^+^-N was 79.31% when the pH value of the solution was 7, and the minimum removal rate was 35.71% when the pH value of the solution was 5 (*p* < 0.05). At a pH value above neutral, the carboxyl group was ionized by providing a hydrogen ion from its hydroxide group, while a hydroxyl group was easily deprotonated to attract positively charged cations, thereby obtaining the capacity to attract negatively charged cations (Badsha et al., 2021). When pH < 7.2, the carboxyl group remained neutral. However, when pH equaled 7.2, the carboxyl group released H^+^ and had negative charges (R-COO^−^; Shalla et al., 2019). In this research, BS was an aerobic denitrifying bacterium with a rapid short-range denitrification capacity for the accumulation of NO_2_^−^-N. The bacterial colony was the dominant colony screened from the actual water sample whose pH value was 7.3, and therefore the removal rate of NO_3_^−^-N reached the maximum at neutral condition. When the water sample was acidic, the adsorption of composite materials was dominant and the growth of BS was weakened. Researches have shown that exposure to extreme pH conditions could adversely affect the reusability of hydrogels in continuous cycles, which was possibly due to morphological damage (Rehman et al., 2016). Therefore, neutral conditions were conducive to the growth of aerobic denitrifying bacteria in PVA/SA/ABC@BS, thus maintaining high decontamination efficiency.

### 3.8. Evaluation on reusability of PVA/SA/ABC@BS

**Figure 8:**
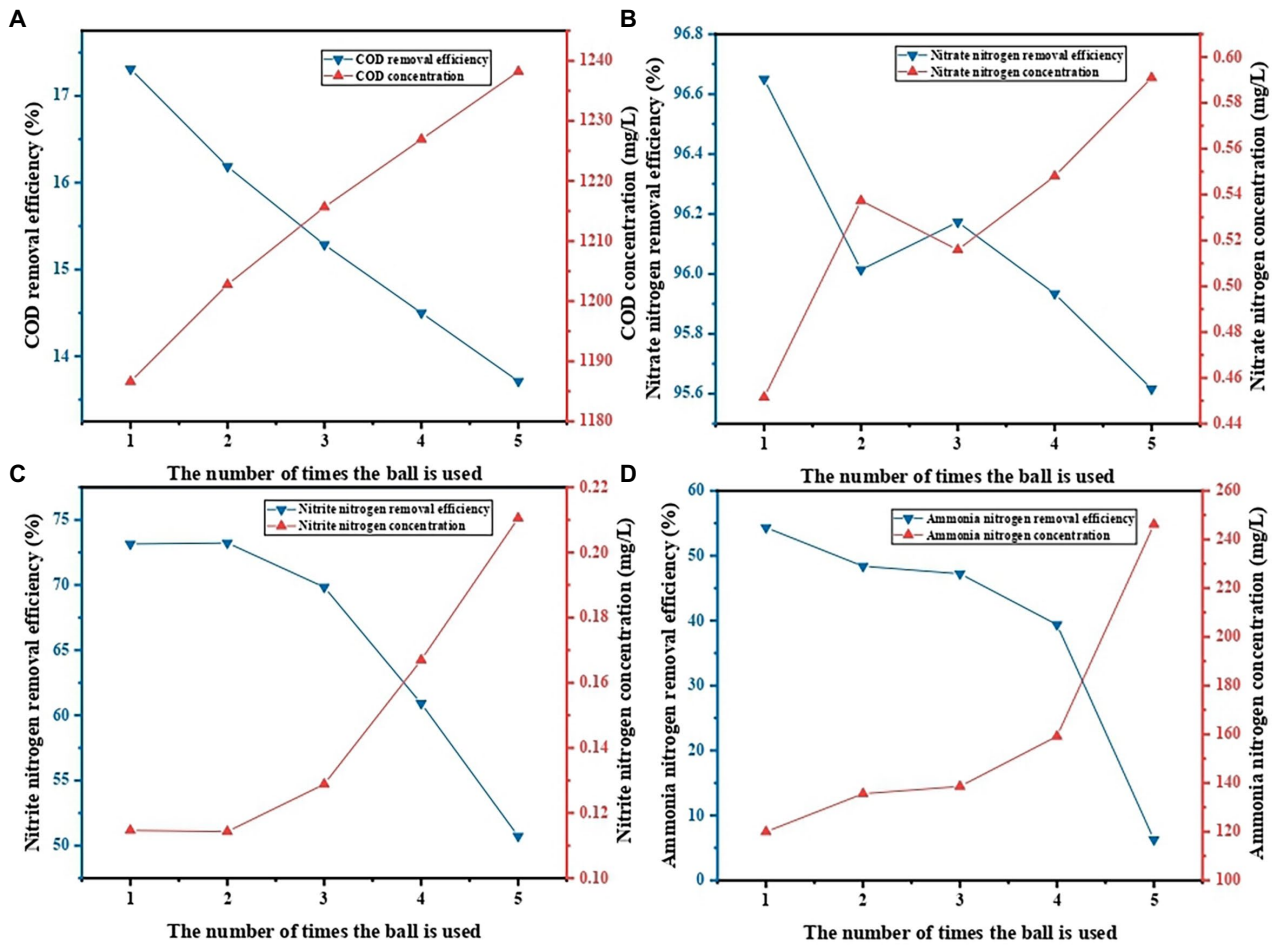
Effect of the number of cycles on contaminant removal: **(A)** COD removal, **(B)** NO_3_^−^-N removal, **(C)** NO_2_^−^-N removal, and **(D)** NH_4_^+^-N removal.

The PVA/SA/ABC@BS prepared in this research still had a high decontamination efficiency after being reused for 5 times. During the decontamination process, pollutants accumulated on the hydrogel until it was completely saturated. The depleted material could be burned or buried in landfills, but it would bring a series of environmental problems (Khan and Lo, 2016). Another environmentally friendly and economical option was to reuse the depleted hydrogel after being recycled (Crini, 2006). Figure 8 shows the treatment effect of leachate during the recycling of PVA/SA/ABC@BS. The results showed that the removal rate of pollutant was the highest after the PVA/SA/ABC@BS was used in the first batch. After repeated use of PVA/SA/ABC@BS in 5 batches and treatment of leachate for 24 h, the removal rate of NO_3_^−^-N reached higher than 95%. This indicates that PVA/SA/ABC@BS had cyclic desorption and adsorption capacity and had excellent reusability. PVA/SA/ABC@BS had certain particle sizes and mechanical properties while retaining the adsorption capacity of biochar, thus increasing its recycling convenience. Therefore, choosing PVA/SA/ABC@BS to treat high concentration organic wastewater could effectively reduce the cost for the treatment of unit volume of wastewater.

## 4. Conclusion

In this work, the aerobic denitrifying bacteria belonging to *Acinetobacter* were successfully immobilized in the gel bead prepared by PVA, SA, and ABC. The introduction of ABC can increase the specific surface area of the gel bead and the number of pore channels inside the gel bead. There were granular bulges on the surface of the gel bead introduced with BC. Under the preparation conditions of 1 g of SA, 100 ml (w/v, 1%) of PVA and 2 g of ABC, the gel beads had excellent mass transfer performance, good bead-forming efficiency, good mechanical strength, and excellent chemical stability. When the time period for coupling gel beads and functional bacteria was 72 h, the effects for the removal of COD and NO_2_^−^-N were the best, and the removal rates were 36.64 and 43.92%, respectively. When the coupling time period was 120 h, the effects for removal of NH_4_^+^-N and NO_3_^−^-N were the best, and the removal rates were 38.08 and 89.40%, respectively. The removal rates of NO_3_^−^-N, NO_2_^−^-N, and NH_4_^+^-N by type II PVA/SA/ABC@BS gel beads were slightly higher than those by type I PVA/SA/ABC@BS gel beads. The removal rate of COD by type II PVA/SA/ABC@BS gel beads was much lower than that by type I PVA/SA/ABC@BS gel beads. When the dosage of PVA/SA/ABC@BS was 0.017 g/ml, the removal rates of NO_3_^−^-N and NH_4_^+^-N were the highest, which were 98.70 and 59.40%, respectively. The concentration of pollutants and dosage of immobilized beads had a little influence on the removal of NO_3_^−^-N while pH had a certain influence on the removal of NO_3_^−^-N. The removal rates of NO_3_^−^-N all reached higher than 95% after repeated use of the immobilized beads in 5 batches, which showed that PVA/SA/ABC@BS had excellent reusability.

## Data availability statement

The data analyzed in this study are subject to the following licenses/restrictions: the data presented in this study are available on request from the corresponding author. Requests to access these datasets should be directed to JS, songjianyang66@163.com.

## Author contributions

JS: conceptualization, data curation, investigation, supervision, funding acquisition, and writing—original draft. ML: investigation, methodology, and writing—review and editing. CW: conceptualization, methodology, and writing—review and editing. YF: conceptualization and writing—review and editing. YL, YW, and WZ: validation and writing—review and editing. HL: writing—review and editing. HW: funding acquisition and writing—review and editing. All authors contributed to the article and approved the submitted version.

## Funding

This work was financially supported by the Henan Key Laboratory of Industrial Microbial Resources and Fermentation Technology, Nanyang Institute of Technology (HIMFT20200205), the Scientific and Technological Projects of Henan Province (212102310278), the Scientific and Technological Projects of Nanyang City (KJGG034), the National Natural Science Foundation of China (52170049), the Doctoral Research Start-up Fund Project of Nanyang Institute of Technology (510161), and the Interdisciplinary Sciences Project, Nanyang Institute of Technology.

## Conflict of interest

The authors declare that the research was conducted in the absence of any commercial or financial relationships that could be construed as a potential conflict of interest.

## Publisher’s note

All claims expressed in this article are solely those of the authors and do not necessarily represent those of their affiliated organizations, or those of the publisher, the editors and the reviewers. Any product that may be evaluated in this article, or claim that may be made by its manufacturer, is not guaranteed or endorsed by the publisher.
